# Interoception in functional motor symptoms and functional seizures: Preliminary evidence of intact accuracy alongside reduced insight and altered sensibility^☆^

**DOI:** 10.1016/j.brat.2023.104379

**Published:** 2023-09

**Authors:** L.S. Merritt Millman, Eleanor Short, Biba Stanton, Joel S. Winston, Timothy R. Nicholson, Mitul A. Mehta, Antje A.T.S. Reinders, Mark J. Edwards, Laura H. Goldstein, Anthony S. David, Matthew Hotopf, Trudie Chalder, Susannah Pick

**Affiliations:** a Institute of Psychiatry, Psychology, and Neuroscience, King's College London, UK; b University College London, Institute of Mental Health, UK

**Keywords:** Interoception, Functional neurological disorder, Pathophysiology, Bodily awareness, Non-epileptic seizures, Metacognition

## Abstract

Altered interoception may be a pathophysiological mechanism in functional neurological disorder (FND). However, findings have been inconsistent across interoceptive dimensions in FND including functional motor symptoms (FMS) and seizures (FS). Here, individuals with FMS/FS (n = 17) and healthy controls (HC, n = 17) completed measures of interoceptive accuracy and insight (adapted heartbeat tracking task [HTT] with confidence ratings), a time estimation control task (TET) and the Multidimensional Assessment of Interoceptive Awareness–2 (MAIA-2) to assess interoceptive sensibility. The groups did not differ in interoceptive accuracy (*p* = 1.00, *g* = 0.00) or confidence (*p* = .99, *g* = 0.004), although the FMS/FS group displayed lower scores on the “Not-Distracting” (*p* < .001, *g* = 1.42) and “Trusting” (*p* = .005, *g* = 1.17) MAIA-2 subscales, relative to HCs. The groups did not differ in TET performance (*p* = .82, *g* = 0.08). There was a positive relationship between HTT accuracy and confidence (insight) in HCs (*r* = .61, *p* = .016) but not in FMS/FS (*r* = 0.11, *p* = .69). HTT confidence was positively correlated with MAIA-2 “Self-Regulation” (*r* = 0.77, *p* = .002) and negatively correlated with FND symptom severity (*r* = −0.84, *p* < .001) and impact (*r* = −0.86, *p* < .001) in FMS/FS. Impaired interoceptive accuracy may not be a core feature in FMS/FS, but reduced insight and altered sensibility may be relevant. Reduced certainty in self-evaluations of bodily experiences may contribute to the pathogenesis of FND symptoms.

## Highlights

• Intact interoceptive accuracy in functional motor symptoms/seizures (FMS/FS).
• Potentially reduced interoceptive insight in FMS/FS versus healthy controls (HC).
• Lower levels of “Trusting” and “Not-Distracting” in FMS/FS compared to HC.
• Elevated “Self-regulation” positively related to interoceptive confidence in FMS/FS.
• Elevated symptom severity/impact negatively related to interoceptive confidence.

## 1 Introduction

Functional neurological disorder (FND) is a condition involving the experience of neurological (seizures, sensory and/or motor) symptoms which are clinically distinguishable from those caused by identifiable neuropathology (APA, 2013; Drane et al., 2021). The mechanisms and aetiology underlying FND remain incompletely understood; however, there have been considerable advances in pathophysiological FND research in recent years, with several possible mechanistic processes highlighted across explanatory models, including disrupted attention, emotional processing, and interoception (Drane et al., 2021; Pick et al., 2019).

Interoception, the sense of awareness of the internal state of the body, is central to the understanding and experience of oneself (Tsakiris, 2018). It is a multidimensional construct including interoceptive accuracy, insight, and sensibility (Khalsa et al., 2018; Box 1).

Box 1 **Definitions (Khalsa et al., 2018)**

***Interoceptive accuracy:*** Correct and precise monitoring of changes in internal bodily states. E.g., degree of accuracy on performance in heartbeat detection tasks.

***Interoceptive insight:*** Metacognitive assessment of performance or experience. E.g., correspondence between accuracy and confidence ratings on heartbeat detection tasks.

***Interoceptive sensibility:*** An individual's perception of their tendency to attend to/focus on internal bodily states in daily life. E.g., Reflections on autobiographical experiences of bodily states over broad time spans using interviews or self-report questionnaires.

Alt-text: Box 1

Models of FND have suggested that discrepancies between top-down and bottom-up processing in the brain and body play a role in the generation of altered interoception and motor or sensory disturbances in FND (Brown & Reuber, 2016; Edwards et al., 2012; Pick et al., 2019; Van den Bergh et al., 2017). Pick et al. (2019), for example, suggest that diminished awareness (interoception) of bodily affective responses (i.e., autonomic arousal) may be an important feature in FND, potentially contributing to impaired top-down regulation of these bodily states and thereby allowing them to exert a disproportionate and disruptive influence on awareness and cognitive/motor control.

Preliminary neuroimaging data further suggest that the insula, a key brain area involved in interoception, may be structurally and/or functionally divergent and linked with alterations in interoceptive processing in FND samples (Pick et al., 2019; Sojka et al., 2020). More specifically, individual differences in interoceptive accuracy and interoceptive trait prediction error in people with functional movement disorders (FMD) have been associated with white matter pathways originating from the insula (Sojka et al., 2020). Reduced insular activation has also been seen during the processing of emotional images and facial expressions in both FMD and functional seizures (FS) (Espay et al., 2018; Szaflarski et al., 2018), and is associated with both alexithymia (Sojka et al., 2019) and symptom severity in FND (Perez, Matin, et al., 2017), suggesting that insular alterations may encourage prediction errors and impair one's ability to accurately perceive bodily signals (Sojka et al., 2020).

Differences in dimensions of interoception have also previously been linked with a range of clinical characteristics in FND including trauma (Pick et al., 2020), emotional processing differences (alexithymia: Demartini et al., 2019), somatoform and psychological dissociation (Koreki et al., 2020; Pick et al., 2020), and indices of clinical severity/complexity (physical symptom burden, depression, anxiety: Koreki et al., 2020; Pick et al., 2020; Ricciardi et al., 2021). These findings suggests that interoception may be connected to both aetiological factors and mechanisms in FND, with the possibility that interoceptive differences may be the link between some of these factors (Koreki et al., 2020).

Previous studies of cardiac *interoceptive accuracy* in patients with FND have provided inconsistent findings. Compared to healthy controls, reduced interoceptive accuracy, as measured with the Heartbeat Tracking Task (HTT; Schandry, 1981), has been seen in some samples with functional motor symptoms (FMS) (Demartini et al., 2019; Ricciardi et al., 2016, 2021), FS (Koreki et al., 2020), and mixed FND symptoms (Williams et al., 2021), but not in others (FS: Jungilligens et al., 2020; mixed symptoms: Pick et al., 2020). Although Pick et al. (2020) did not find accuracy to be reduced at baseline, accuracy was impaired after the induction of a dissociative state, aligning with other findings (Koreki et al., 2020). The intact baseline interoceptive accuracy observed by Pick et al. (2020) was coupled with reduced confidence ratings in the FND group, indicating a potential difference in metacognitive *interoceptive insight* characterised by underestimation of their performance. However, findings in the insight dimension are also variable (Ricciardi et al., 2021). These inconsistencies further extend to the dimension of self-reported trait *interoceptive sensibility*. Compared to HCs, heightened sensibility was reported by a FS sample (Koreki et al., 2020), although reduced sensibility was reported in a FMD sample (Ricciardi et al., 2021), specifically a reduction in ability to recognize illness signals and predict bodily reactions. In mixed FND samples (Pick et al., 2020), differences between cases and controls also suggest altered sensibility in FND in the form of a greater tendency to distract from unpleasant or uncomfortable bodily sensations as well as reduced subjective trust and safety in the body (Pick et al., 2020).

The discrepancies in results could be due to inconsistent measurement of interoceptive dimensions and variable control of known confounds of the HTT (Murphy et al., 2018; Palmer, Ainley, & Tsakiris, 2019; Ring & Brener, 1996) including body mass index (BMI), knowledge of own heart rate, and time estimation abilities, as well as an overwhelming focus on interoceptive accuracy, with measures of interoceptive insight or sensibility less commonly included. The use of standard HTT instructions alongside pulse oximeters or worn sensors that may facilitate task performance (Murphy et al., 2019) may also account for some discrepancies.

Given the possibility that disrupted interoception may play a role in the development or maintenance of FND symptoms, further research is warranted to identify whether interoceptive accuracy is impaired in specific FND subgroups. Furthermore, closer examination of the role of interoceptive insight and sensibility in FND is needed to identify the most notable differences in these dimensions in FND subgroups. Given the variable control of confounding factors in previous studies, it is important to assess whether performance on the HTT in FND samples is reliably affected by common confounds, such as BMI, previous knowledge of heart rate, and time estimation abilities.

### 1.1 Aims and hypotheses

As part of a larger pilot project using multimodal research methods to examine psychobiological causes and mechanisms in two common subgroups of FND (FMS/FS), the aim of this experiment was to assess interoception in this population across three dimensions. We aimed to pilot the procedures and test the hypotheses that interoceptive accuracy and/or insight would be significantly reduced in the FMS/FS group compared to HCs (Pick et al., 2019), whilst evaluating the potential influence of several possible confounds, including time estimation, possible device-related issues (Desmedt et al., 2020), BMI, general cognitive functioning, and comorbid psychological and physical symptoms. Based on previous findings (Pick et al., 2020), we predicted that the FMS/FS group would report significant alterations in interoceptive sensibility on two or more subscales of the Multidimensional Assessment of Interoceptive Awareness–2 (MAIA-2) compared to HCs.

An additional aim was to better understand how interoception is related to a range of relevant clinical characteristics in FMS/FS. If interoceptive differences are, in fact, underlying mechanisms in FND, this would predict significant correlations between FND symptom severity and impact and interoception (Koreki et al., 2020; Ricciardi et al., 2021). Given that alexithymia involves difficulties identifying and describing emotions, elevated alexithymia, as seen in FND (Demartini et al., 2014), may be linked to differences in interoception due to an impaired processing of emotional bodily states (Demartini et al., 2019; Pick et al., 2019). Similar difficulties have been identified in autistic spectrum disorder, suggesting that elevated autistic traits in FND may also be tied to reductions or alterations in interoception (Gonzalez-Herrero et al., 2022). Associations between elevated psychological distress (depression and anxiety) and altered interoceptive accuracy and sensibility (Demartini et al., 2019; Pick et al., 2020) are also present in FND, and these variables may reciprocally influence one another. Previous research has also made apparent the links between dissociation and interoception in FND (Jungilligens et al., 2020; Koreki et al., 2020; Pick et al., 2020): dissociative experiences are common in FND and negatively correlate with, and can directly impair, interoceptive accuracy (Pick et al., 2020), thus informing the prediction that there would be significant relationships between dissociation and dimensions of interoception. Exploratory correlational analyses examined associations between interoceptive dimensions and these clinically relevant variables.

## 2 Methods

### 2.1 Participants

Seventeen patients with FMS/FS and 17 healthy controls were included. The sample size was deemed appropriate given the goals of the broader pilot project in which this study was conducted. Patients with FMS/FS were recruited online via mailing lists, social media, and advertisements circulated by charitable patient support websites (FND Action, FND Hope UK). Healthy controls were recruited through local community websites (South London Facebook and Gumtree).

No participants included in this study overlapped with the samples reported in earlier studies from this group (Pick et al., 2020). The study was approved by the King's College London Health Faculties High-Risk Research Ethics Sub-Committee (ref: HR/DP-21/22–28714) and conforms to the World Medical Association Declaration of Helsinki. Data collection took place between July and October 2022.

Participants were between the ages of 18–65 years old, with normal or corrected eyesight and fluency in English. Participants in the FMS/FS group were required to have a primary diagnosis of FND (DSM-5) (APA, 2013), with FMS and/or FS as their primary complaint, confirmed with medical documentation checked by the principal investigator (SP) and in some cases a Consultant Neurologist (BS). The exclusion criteria were: major comorbid cardiovascular or neurological disorder, active severe psychiatric disturbance, or physical symptoms/disability that would confound the findings or impair task performance, taking medications affecting cardiovascular functioning (e.g., beta-blockers) or attention and concentration (i.e., daily/daytime opioids, barbiturates), and pacemakers. Healthy control participants were excluded if they disclosed lifetime functional neurological symptoms or reported the presence of an active major physical or mental health disorder.

### 2.2 Procedure

After written, informed consent was obtained, a comprehensive screening interview was conducted remotely, to obtain data regarding sociodemographic characteristics and medical history. Participants were asked if they had any prior knowledge of their own heart rate (e.g., from a wearable device) and for their height and weight so BMI could be calculated. A tailored structured clinical interview (SCID-5-RV) (First, Williams, Karg, & Spitzer, 2015) was administered remotely by SP to assess the possible presence of mental health disorders relevant to the eligibility criteria (e.g., active psychosis, severe affective disorder, substance/alcohol dependence).

Eligible participants were sent a set of self-report questionnaires to complete online via Qualtrics (www.qualtrics.com) within 48 h prior to attending the laboratory session. The laboratory session took place in a quiet testing room at the Institute of Psychiatry, Psychology and Neuroscience. Within this session, participants completed the HTT (Schandry, 1981) and the TET, alongside other experimental and neurocognitive tasks (reported elsewhere). Upon completion, participants were compensated with a £50 shopping voucher.

### 2.3 Measures

The *Heartbeat Tracking Task* (HTT) (Schandry, 1981) was included as a measure of interoceptive accuracy and insight, administered with E-Prime experimental software (Psychology Software Tools, Inc.). As recommended to control for device-related confounds (Murphy et al., 2019), electrocardiography (ECG) was used to record heartbeats throughout the task. Participants were seated at a table in front of a computer and asked to attend to and count their own heartbeats during three randomised intervals (25, 35, and 45s). Adapted instructions were used, specifically asking participants to only count the heartbeats they could feel (Desmedt et al., 2020). Participants were also explicitly asked not to count seconds or take their own pulse, or use any device to measure their heartbeats. Thirty second rest periods between each of the heartbeat counting trials were provided. The start and end of each trial were indicated on screen. Immediately after each trial, participants were asked to manually report the number of perceived heartbeats, followed by rating their confidence in their answer (0–10, low-high certainty). Participants did not receive any feedback on task performance and were unaware of the duration of the trials. A practice trial was completed prior to starting the experimental trials.

The *Time Estimation Task* (TET) was used as a control task. In the TET, participants were asked to count seconds during three randomised intervals (23, 37, 42s), separated by rest periods of 10 s. The start and end of each trial was indicated on screen. Immediately after each trial, participants were asked to report manually the number of seconds they had counted and then to rate their confidence (0–10, low-high certainty). Participants did not receive any feedback on task performance.

The *Multidimensional Assessment of Interoceptive Awareness – second edition* (MAIA-2) (Mehling et al., 2018) is a 37-item self-report questionnaire assessing trait-level abilities relating to interoceptive sensibility and recognition of bodily experiences across eight dimensions: Noticing (4 items; α = 0.74), Not-Distracting (6 items; α = 0.89), Not-Worrying (5 items; α = 0.68), Attention Regulation (7 items; α = 0.89), Emotional Awareness (5 items; α = 0.88), Self-Regulation (4 items; α = 0.78), Body Listening (3 items; α = 0.73), Trusting (3 items; α = 0.82). Each question is scored on a Likert-scale from 0 (“never”) to 5 (“always”).

A bespoke Functional Neurological Symptoms Questionnaire was designed for this study to assess the presence, frequency, severity, and impact of FND symptoms (Supplementary File 1). Participants are asked to report the presence/absence (Yes/No), frequency (constant/daily/weekly/less than weekly), severity (1 = “Symptom not present” to 7 = “Very severe”), and impact (1 = “No impact at all” to 7 = “Very severe impact”) of FND symptoms within the past week.

### 2.4 Analysis

All data were analysed using *R* (Version 4.1.0, 2021). Missing data on the self-report measures were addressed as follows: for participants missing 20% or less of a given scale (or subscale), the missing item/s were imputed with the mean of that individual's scores for the scale (or subscale). If more than 20% of the data for one scale or subscale was missing, the participant was excluded from that analysis. This resulted in 0–15% of participants being excluded from the MAIA-2 subscales. Due to technical issues, two participants were missing the TET (1 FMS/FS, 1 control) and three participants were missing the HTT (1 FMS/FS, 2 controls). Normality was evaluated with Shapiro-Wilk test and QQ-plots for each variable. Categorical variables were analysed with Fisher's exact tests and continuous variables were analysed with independent samples Welch's t-tests, with Hedges' g (Hedges & Olkin, 1985) as the effect size. To calculate interoceptive accuracy, the proportional discrepancy between the actual and perceived number of heartbeats was calculated using the following formula: 1/3 ∑ [(1 − (|actual heartbeats – perceived heartbeats|/actual heartbeats). This resulted in an accuracy error index with values closer to 1 reflecting a lower discrepancy and superior interoceptive accuracy (Schandry, 1981). The same formula was used for the TET: 1/3 ∑ [(1 − (|actual seconds – perceived seconds|/actual seconds). To examine interoceptive insight, we used Pearson's correlations to assess the degree of association between HTT accuracy and confidence ratings. Bonferroni corrections were used in the case of multiple tests conducted on related variables (e.g., subscales of questionnaires, dependent variables in the behavioural tasks). Exploratory Pearson's or Spearman's correlations were computed to assess associations between interoceptive dimensions and clinical characteristics in the FMS/FS group.

## 3 Results

### 3.1 Participant characteristics

The characteristics of the sample are detailed in Table 1. The groups were comparable in age, gender, BMI, and intellectual functioning. The FMS/FS group reported additional functional symptoms including dizziness, cognitive difficulties, sensory, and speech/swallowing difficulties. The FMS/FS group had a significantly higher resting heart rate, and significantly more participants in the FMS/FS group disclosed previous knowledge of their heart rate, were more likely to be taking medication, and were experiencing a comorbid mental or physical health disorder. Details of medications, physical health diagnoses and mental health diagnoses are reported in Supplementary Table 1.

**Table 1:** Demographic information as a function of group.

Variable	FMS/FS (*n* = 17)	Control (*n* = 17)			
	M (*SD*)	M (*SD*)	*t* (*df*)	*p*	*g*
**Age**	36.5 (10.6)	39.0 (11.0)	.67 (31.95)	.51	.23
**BMI**	28.4 (7.5)	25.4 (5.32)	−1.35 (28.87)	.19	.45
**Resting heart rate**	81.6 (11.9)	72.7 (8.28) (n = 16)	−2.49 (28.59)	.02	.86
**FSIQ**	102 (14.8)	107 (9.25)	1.08 (26.84)	.29	.41

	***n (%)***	***n (%)***	***p***
**Gender (% female)**	13 (76)	13 (76)	.656 (Fisher's)
**Knowledge of heart-rate (% yes)**	12 (71)	3 (18)	.005 (Fisher's)
**Mental health diagnosis (% yes)**	10 (59)	1 (6)	.001 (Fisher's)
**Physical health diagnosis (% yes)**	12 (71)	4 (24)	.007 (Fisher's)
**Medication (% yes)**	16 (94)	5 (29)	<.001 (Fisher's)
**Average symptom severity and impact (1–7): M (*SD*)**	Severity = 4.09 (0.96)		
	Impact = 4.10 (0.85)		
**Self-reported FND symptoms: n (%)**	Seizures = 7 (41)		
	Motor = 17 (100)		
	Dizziness = 14 (82)		
	Cognitive = 14 (82)		
	Speech/swallowing = 9 (53)		
	Sensory = 17 (100)		
	Other = 10 (59)		
	Multiple = 17 (100)		

*Notes.* FMS/FS = functional motor symptoms/functional seizures; FSIQ = Full scale intelligence quotient; M = mean; SD = standard deviation.

### 3.2 Interoceptive accuracy and insight

The two groups did not differ in accuracy or confidence ratings on the HTT (all negligible effect sizes, see Table 2). These results held when the analyses were re-run excluding cases where data was missing for the HTT (FMS/FS: n = 16, HC: n = 14; all *p*-values >.85; Supplementary Table 2).

**Table 2:** Interoception: accuracy, insight, and sensibility.

Variable	FMS/FS	Control	*t* (*df*)	*p*	*g*	*95*% CI
	M (*SD*) [*n*]	M (*SD*) [*n*]				
**HTT Accuracy**	.536 (.239) [16]	.536 (.257) [15]	.003 (28.45)	1.00	.00	−.18, .18
**HTT Confidence**	4.92 (2.46) [16]	4.93 (2.74) [15]	.018 (28.16)	.99	.004	−1.90, 1.93
**TET Accuracy**	.770 (.136) [16]	.781 (.141) [16]	.230 (29.97)	.82	.08	−.09, .11
**TET Confidence**	6.83 (2.04) [16]	6.75 (1.63) [16]	−.128 (28.58)	.90	.04	−1.42, 1.25
**MAIA Noticing**	3.42 (1.02) [16]	2.94 (.96) [13]	−1.30 (26.36)	.21	.47	−1.24, .28
**MAIA-2 Not-Distracting**	1.28 (.88) [16]	2.46 (.71) [14]	4.08 (27.83)	<.001	1.42	.59, 1.78
**MAIA-2 Not-Worrying**	2.86 (.77) [16]	2.77 (.86) [15]	−.30 (28.23)	.76	.11	−.69, .51
**MAIA-2 Attention Regulation**	2.52 (.84) [15]	2.66 (1.24) [15]	.37 (24.59)	.71	.13	−.65, .94
**MAIA-2 Emotional Awareness**	2.93 (1.14) [15]	2.74 (1.41) [15]	−.41 (26.86)	.69	.14	−1.15, .77
**MAIA-2 Self-Regulation**	2.02 (.99) [14]	2.67 (1.04) [15]	1.72 (26.99)	.10	.62	−.12, 1.42
**MAIA-2 Body Listening**	2.31 (1.11) [12]	2.00 (1.01) [15]	−.74 (22.53)	.47	.28	−1.16, .55
**MAIA-2 Trusting**	2.26 (1.44) [14]	3.64 (.78) [15]	3.17 (19.7)	.005	1.17	.47, 2.29

*Notes.* HTT = Heartbeat Tracking Task; TET = Time Estimation Task; M = mean; MAIA-2 = Multidimensional Assessment of Interoceptive Awareness-2; SD = standard deviation.

There was a strong positive relationship between HTT accuracy and confidence ratings (interoceptive insight) in the control group but not in the FMS/FS group (Table 3), although the difference between coefficients was a non-significant trend (*z* = 1.59, *p* = .068).

**Table 3:** Significant exploratory correlations.

Variable	FMS/FS (n = 17)			Control (n = 17)		
	*r*	*p*	95% CI	*r*	*p*	95% CI
**HTT accuracy**						
HTT confidence	.11	.69	−.41, .57	.61	.016	.14, .86
Gender	.02	.94	−.48, .51	−.60	.017	−.85, −.13

**HTT confidence**						
MAIA-2 Self-Regulation	.77	.002	.37, .93	.29	.34	−.31, .72
FND symptom severity	−.84	<.001	−.94, −.58			
FND symptom impact	−.86	<.001	−.95, −.63			

*Notes.* CI = confidence interval; HTT = Heartbeat Tracking Task; MAIA-2 = Multidimensional Assessment of Interoceptive Awareness-2; FND = functional neurological disorder.

### 3.3 Self-reported interoceptive sensibility

Compared to controls, the FMS/FS group displayed significantly lower scores on the “Not-Distracting” (e.g., ‘I distract myself from sensations of discomfort’, ‘I try to ignore pain’ – reverse scored) and “Trusting” (e.g., ‘I am at home in my body’, ‘I trust my body sensations’) subscales of the MAIA-2 (Table 2), both withstanding a Bonferroni-adjusted alpha and with large effect sizes. There were no significant between-group differences on the other MAIA-2 subscales.

### 3.4 Exploratory analyses

Table 3 presents the statistical values for the significant exploratory analyses described below. Non-significant findings are detailed in Supplementary Tables 4 and 6

#### 3.4.1 Examining the influence of possible confounding variables

The two groups did not differ in accuracy or confidence ratings on the TET (all negligible effect sizes, Table 2). These results held when the analyses were re-run excluding cases where data was missing for the TET (FMS/FS: n = 16, HC: n = 14; all *p*-values >.83; Supplementary Table 2). No association was seen between TET accuracy and confidence in either group (Supplementary Table 4).

There was no association in either group between HTT and TET accuracy or HTT and TET confidence ratings (Supplementary Table 4). We ran correlations between key interoceptive outcomes (HTT accuracy and confidence, MAIA-2 Trusting and Not-Distracting) and a range of potential confounds, including: BMI, PHQ-9 (depression), PHQ-15 (physical symptoms), GAD-7 (anxiety), age, gender, intellectual functioning, self-reported previous knowledge of heart rate, baseline heart rate, medication, current physical health diagnoses, and current mental health diagnoses. There were no significant correlations (Supplementary Table 4) except for a strong, significant association between gender and HTT accuracy in HCs, wherein female participants displayed greater HTT accuracy (Table 3).

#### 3.4.2 Correlations among interoceptive dimensions

There was a strong, significant relationship between HTT confidence ratings and the “Self-Regulation” subscale of the MAIA-2 in the FMS/FS group (Table 3), withstanding a Bonferroni correction (adjusted alpha = .006). No other significant associations were seen between interoceptive accuracy or confidence and MAIA-2 scores in either group (Supplementary Table 4).

#### 3.4.3 Correlations between interoception and relevant clinical variables

In the FMS/FS group, there were significant correlations between self-reported FND symptom severity and HTT confidence scores, and FND symptom impact and HTT confidence scores (Table 3), both with large effect sizes and withstanding Bonferroni corrections. There were no other significant associations between interoceptive accuracy, confidence, or sensibility (MAIA-2) and any other clinical/background variables in the FMS/FS group (SDQ-20, TAS-20, AQ, MDI [Supplementary Tables 5 and 6]).

## 4 Discussion

This study aimed to elucidate the possible role of interoceptive processing in FMS/FS, testing the hypotheses that interoceptive accuracy and insight would be reduced, and interoceptive sensibility would be significantly altered in FMS/FS compared to HCs. A further aim was to explore how interoception may be related to a range of relevant aetiological factors and clinical characteristics in FMS/FS. These results present the possibility that there may be a separation between interoceptive accuracy and confidence in FMS/FS, suggesting a potential metacognitive difference characterised by reduced certainty/confidence in one's own interoceptive perceptions. Furthermore, a reduced subjective sense of trust within the body and a tendency towards distracting from uncomfortable bodily sensations seen in FMS/FS may also be linked to a reduced confidence in self-evaluations of bodily experience, possibly reinforcing FND symptoms.

There was no evidence of impaired interoceptive accuracy in the FMS/FS group compared to controls, aligning with previous work by Pick et al. (2020) and Jungilligens et al. (2020). Whilst our sample size limited the statistical power to detect significant between-group differences, the effect sizes indicated that there was no group effect on HTT accuracy. This is contrary to other studies reporting reduced interoceptive accuracy in more specific FND subgroups, including those with FMS (Demartini et al., 2019; Ricciardi et al., 2016, 2021) and FS (Koreki et al., 2020), and does not support the proposal that altered interoception is a core feature in FND. Nevertheless, this study examined only cardiac interoception, at rest. It remains a possibility that FND is associated with impairments of interoception in other bodily domains, or with state-dependent interoceptive differences, such as during dissociative states or acute affective arousal (Pick et al., 2019, 2020). The differential results across studies could also be due to variations in samples regarding symptom types, the presence of comorbid mental health diagnoses, modest sample sizes, and inconsistent measurement and inclusion of potential confounds of heartbeat tracking tasks.

The current study did not reveal generally reduced interoceptive confidence ratings in the FMS/FS group. However, while there was a strong, positive relationship between HTT accuracy and confidence in controls suggestive of adequate interoceptive insight, this association was not seen in those with FMS/FS. This potential disconnect between actual performance and subjective confidence on the HTT in the FMS/FS group suggests that FMS/FS may be associated with less accurate self-evaluations of interoceptive performance (i.e., reduced interoceptive insight). Similarly, this sample displayed discrepancies between objective and subjective neurocognitive functioning, including tests of attention and executive functions, pointing towards possible generalised deficits in metacognition in this sample (Pick et al., under review, Journal of Clinical and Experimental Neuropsychology). This aligns with other research proposing metacognitive deficits in FND, specifically impaired metacognition in functional cognitive disorders (Bhome et al., 2022; Larner, 2021; Teodoro et al., 2023). Further research is required to better understand local versus global metacognitive abilities in these disorders, and the ways in which impaired metacognition may contribute to FND symptoms.

Though a higher proportion of this FMS/FS group reported previous knowledge of their heart rate, a higher resting heart rate, current medication, and physical and mental health diagnoses, these variables, alongside BMI, showed no significant relationship with any of the interoceptive constructs tested here, suggesting that these potential confounds did not meaningfully affect our results. Gender was also nonsignificant in relation to the interoceptive constructs tested in the FMS/FS group, although there was a significant association between gender and HTT accuracy in controls, wherein female participants displayed greater accuracy. This is consistent with some previous research revealing a female bias towards interoceptive awareness, potentially due to elevated cognitive empathy and emotion recognition (Grabauskaitė et al., 2017).

In the present study, the two participant groups did not differ, with negligible effect sizes, in TET accuracy or TET confidence ratings. Unlike some previous studies using the HTT alongside the TET (Palmer, Ainley, & Tsakiris, 2019; Ring & Brener, 1996; Ring et al., 2015), there was no association in either group between HTT and TET accuracy or confidence, suggesting that performance on the HTT was unlikely to have been influenced by time estimation abilities.

Altered self-reported interoceptive sensibility was seen in this FMS/FS sample across two subscales of the MAIA-2, both with large effect sizes. These differences in aspects of interoceptive sensibility paired with intact accuracy and confidence point towards the possibility that there is a separation between trait and state measures of interoception in FMS/FS. Individuals with FMS/FS exhibited lower scores on the “Not-Distracting” subscale, indicating an inclination towards ignoring or distracting from uncomfortable or unpleasant bodily sensations, and the “Trusting” subscale, suggesting that they feel less at home in their body, not trusting their bodily sensations, compared to controls (Mehling et al., 2018), which aligns with some previous results on interoceptive sensibility in FND (Pick et al., 2020; Ricciardi et al., 2021). Lower levels of trusting the body may be linked to the reduced interoceptive insight seen in this study, suggesting the possibility of a general reduction in confidence and trust in one's self-evaluations of bodily experiences, which may be present at both local and global levels. However, the finding that individuals with FMS/FS are more likely to distract from uncomfortable or unpleasant bodily sensations is contrary to some models of FND (Edwards et al., 2012; Van den Bergh et al., 2017). The tendencies towards distraction and not trusting the body as seen in this sample may be a secondary consequence of living with a chronic physical illness, but equally could be what reinforces symptoms or makes individuals more susceptible to them. These possibilities are not mutually exclusive and longitudinal studies would be needed to elucidate the direction of these relationships. In future research, the inclusion of clinical control groups, such as individuals with other chronic physical health disorders, will help to establish the specificity of these results.

Exploratory correlations revealed that the “Self-Regulation” subscale (MAIA-2) was strongly positively correlated with HTT confidence in the FMS/FS group, whereas self-reported FND symptom severity and impact were strongly negatively correlated with HTT confidence. Self-regulation requires bringing awareness to the body, using breathing or focusing in on the body to reduce tension or calm the mind. These results imply that those individuals with FMS/FS who were better able to self-regulate were those with higher levels of subjective interoceptive confidence, while individuals experiencing more severe and impactful FND symptoms were those with lower levels of subjective interoceptive confidence. Given the correlational nature of these findings, it is not possible to make inferences regarding the direction of these relationships; it is possible that reduced interoceptive insight could be a predisposing factor for developing more severe FND symptoms, or it may be a direct result of living with severe FND symptoms.

These results have potential implications for treatment, suggesting that interventions targeting aspects of attention and bodily awareness may be particularly beneficial for this population. Therapeutic approaches that encourage a sense of trust and confidence within the body alongside a focus on feelings and interoceptive stimuli, rather than trying to distract from them, could provide benefits in FND. Specific possibilities include body scanning techniques (Gibson, 2019), Somatic Experiencing (Payne et al., 2015), attention training (Wells et al., 1997), and Mindfulness Oriented Meditation (D'Antoni et al., 2022).

### 4.1 Strengths and limitations

Given the aims of this pilot study, the HTT was selected as a feasible, well-established, and widely used measure of interoceptive accuracy and insight (Garfinkel et al., 2015). However, the reliability and validity of the task has been questioned, with suggestions that task performance can be impacted by prior beliefs about one's heart rate or estimating the amount of time that has passed, rather than an accurate perception of heartbeats (Desmedt et al., 2020; Murphy et al., 2019). The use of the HTT with adapted instructions, measurement of self-report BMI and knowledge of heart rate, heart rate measurement via ECG, and inclusion of the TET control task are key strengths of this study that help to minimise the possible influence of estimation abilities other known confounds of the HTT in this study. Nevertheless, future research should aim to include alternative tasks that are not susceptible to these possible confounds. Whilst the results are limited due to the small sample size and modest statistical power, we have presented and considered effect sizes throughout to demonstrate the presence of meaningful differences.

The cross-sectional nature of this experiment does not allow conclusions to be drawn regarding the direction of the relationship between FMS/FS and the interoceptive differences observed. The inclusion of an FND sample experiencing primary FMS/FS alongside other functional neurological symptoms, though a strength in terms of generalizability, is a limitation given the possibility that interoceptive deficits may manifest differently depending on symptom types. Future research should aim to examine interoception in the broader FND population alongside specific symptom subtypes.

It is possible that the anxiety measure included in this study did not assess somatic symptoms of anxiety and could be the reason for the lack of relationship between interoceptive measures and anxiety. Measures more likely to capture somatic anxiety symptoms should be considered in future. It will be particularly useful in future studies to include clinical comparison groups such as those with relevant mental health disorders (depression, anxiety, PTSD) or other chronic physical health disorders such as neurological diseases. Future research should also aim to include a larger sample as well as objectively measure relevant variables (e.g., BMI).

### 4.2 Conclusions

Individuals with FMS/FS may not exhibit consistently reduced interoceptive accuracy, but may experience reduced interoceptive insight alongside altered interoceptive sensibility (“Not-Distracting”, Trusting”). These results provide further evidence that there may be a separation between trait and state interoception in FMS/FS. Our future work will explore the possibility that interoceptive differences in FND may vary in specific FND subgroups, measuring these interoceptive domains with additional paradigms, in larger samples, compared to both healthy and clinical controls. Further research should aim to test the potential benefits of interventions aimed at bodily-focused attention or metacognition in FND.

## Source of funding

The study was supported by a Medical Research Council Career Development Award to SP [MR/V032771/1]. This paper also represents independent research part-funded by the National Institute for Health and Care Research (NIHR) Biomedical Research Centre at South London and Maudsley NHS Foundation Trust and King's College London. The views expressed are those of the authors and not necessarily those of the NHS, the NIHR or the Department of Health and Social Care.

## CRediT authorship contribution statement

**L.S. Merritt Millman:** Data curation, Formal analysis, Writing – original draft, Writing – review & editing. **Eleanor Short:** Data curation. **Biba Stanton:** Methodology, Supervision, Writing – review & editing. **Joel S. Winston:** Methodology, Supervision, Writing – review & editing. **Timothy R. Nicholson:** Methodology, Supervision, Writing – review & editing. **Mitul A. Mehta:** Methodology, Supervision, Writing – review & editing. **Antje A.T.S. Reinders:** Methodology, Supervision, Writing – review & editing. **Mark J. Edwards:** Methodology, Supervision, Writing – review & editing. **Laura H. Goldstein:** Methodology, Supervision, Writing – review & editing. **Anthony S. David:** Methodology, Supervision, Writing – review & editing. **Matthew Hotopf:** Methodology, Supervision, Writing – review & editing, Funding acquisition, Resources. **Trudie Chalder:** Methodology, Supervision, Resources, Funding acquisition, Writing - review & editing. **Susannah Pick:** Funding acquisition, Conceptualization, Methodology, Software, Investigation, Data curation, Resources, Validation, Supervision, Writing – review & editing.

## Declaration of competing interest

The authors declare no conflict of interest.

## Data Availability

Data will be made available on request.
