# Supplementary material for: Interoception in functional motor symptoms and functional seizures: Preliminary evidence of intact accuracy alongside reduced insight and altered sensibility
Source: Behav Res Ther. 2023 Sep;168:104379. doi: 10.1016/j.brat.2023.104379 (PMC10788481; doi:10.1016/j.brat.2023.104379)
Supplement: Multimedia component 1 [file mmc1.docx]

**Supplementary File 1**

**Functional Neurological Symptoms Questionnaire**

Please look at the symptoms in the table below and tell us whether you have experienced these functional neurological symptoms in the **past week.** If you mark yes to indicate that the symptom was present in the past week, please complete the additional columns to tell us **how frequent** the symptoms were, **how severe** (intense) they were, and **how much impact** they had on you.

When rating the average **severity** of symptoms, please choose a number from 1 to 7, where **1=Symptom not present; 2=Minimal; 3=Mild; 4=Moderate; 5=Moderately severe; 6=Severe; 7=Very severe**. When rating the **impact** of symptoms, please choose a number from 1 to 7, where **1=No impact at all; 2=Minimal impact; 3=Mild impact; 4=Moderate impact; 5=Moderately severe impact; 6=Severe impact; 7=Very severe impact**.

| **FND Symptom** | **Present?**  (circle or bold) | **Frequency**  (circle or bold) | **Average severity**  **(1-7)** | **Average impact**  **(1-7)** |
| --- | --- | --- | --- | --- |
| Weakness | Yes / No | Constant / daily / weekly / less than weekly |  |  |
| Tremor | Yes / No | Constant / daily / weekly / less than weekly |  |  |
| Dystonia (muscle spasms / fixed postures) | Yes / No | Constant (1) / daily (2) / weekly (3) / less than weekly (4) |  |  |
| Walking / mobility difficulties | Yes / No | Constant / daily / weekly / less than weekly |  |  |
| Myoclonus (muscle jerks) | Yes / No | Constant / daily / weekly / less than weekly |  |  |
| Seizures* | Yes / No | Number of seizures in the last week: |  |  |
| Numbness (loss of feeling) | Yes / No | Constant / daily / weekly / less than weekly |  |  |
| Visual disturbances | Yes / No | Constant / daily / weekly / less than weekly |  |  |
| Sensitivity to light/sound | Yes / No | Constant / daily / weekly / less than weekly |  |  |
| Dizziness | Yes / No | Constant / daily / weekly / less than weekly |  |  |
| Speech / swallowing difficulties | Yes / No | Constant / daily / weekly / less than weekly |  |  |
| Cognitive difficulties (e.g., brain fog, memory lapses) | Yes / No | Constant / daily / weekly / less than weekly |  |  |
| Other FND symptoms | Details: | Constant / daily / weekly / less than weekly |  |  |

Please tell us which FND symptom(s) is most severe and has the most impact on you:

…............................................................................................................................................

*If you experience FND seizures, do you have warning symptoms? Yes / No

*If you experience warning symptoms before an FND seizure, what is the earliest or most consistent symptom(s) that you experience?

…..................................................................................................................................................................................................................................................................................................

**Supplementary Table 1.** Medications, physical and mental health diagnoses in FND and HCs

| **Variable** | **FND**  **(n = 17)** | **Control**  **(n = 17)** |
| --- | --- | --- |
|  | ***n (%)*** | ***n (%)*** |
| **Mental health diagnosis (% yes)** | 10 (59) | 1 (6) |
| **Seasonal Affective Disorder** |  | 1 (6) |
| **Panic attacks** | 2 (12) |  |
| **Depression** | 8 (47) |  |
| **Post-Traumatic Stress Disorder** | 1 (6) |  |
| **Post-natal depression** |  | 1 (6) |
| **Anxiety** | 7 (41) |  |
| **Borderline Personality Disorder** | 1 (6) |  |
| **Physical health diagnosis (% yes)** | 12 (71) | 4 (24) |
| **Eczema** |  | 1 (6) |
| **Allergies** | 1 (6) |  |
| **Asthma** | 2 (12) | 1 (6) |
| **Hyperthyroidism** |  | 1 (6) |
| **Irritable Bowel Syndrome** | 2 (12) | 1 (6) |
| **Hemicrania continua** | 1 (6) |  |
| **Sciatica** |  | 1 (6) |
| **Endometriosis** | 1 (6) |  |
| **Vasovagal syncope** | 1 (6) |  |
| **Fibromyalgia** | 2 (12) |  |
| **Hepatitis B** | 1 (6) |  |
| **Migraines** | 1 (6) |  |
| **Herpes** | 1 (6) |  |
| **Chronic pain** | 1 (6) |  |
| **Frequent fainting** | 1 (6) |  |
| **Ehlers-Danlos Syndrome** | 1 (6) |  |
| **Medication (% yes)** | 16 (94) | 5 (29) |
| **PPI** | 2 (12) |  |
| **Dopamine agonist** | 1 (6) |  |
| **Anti-convulsant** | 6 (35) |  |
| **Antidepressant** | 10 (59) | 1 (6) |
| **Antipsychotic** | 1 (6) |  |
| **Antihistamine** | 1 (6) | 2 (12) |
| **Botox** | 1 (6) |  |
| **Anti-migraine** | 1 (6) |  |
| **Anti-spasmodic** |  | 1 (6) |
| **Anti-thyroid agent** |  | 1 (6) |
| **Muscle relaxant** | 1 (6) |  |
| **Anti-constipation** | 2 (12) |  |
| **CBD** | 2 (12) |  |
| **Botox** | 1 (6) |  |
| **Paracetamol** | 2 (12) |  |
| **NSAID** | 2 (12) |  |
| **Lipophilic/hydrophilic** | 1 (6) |  |
| **High blood pressure** | 1 (6) |  |
| **Levothyroxine** | 1 (6) |  |
| **Contraceptive** | 1 (6) | 1 (6) |
| **Selenium** |  | 1 (6) |
| **Opiate analgesic** | 4 (24) |  |
| **Anti-asthmatic** | 2 (12) |  |
| **Anxiolytic** | 4 (24) |  |
| **HRT** | 1 (6) |  |
| **NRTI** | 1 (6) |  |
| **Anti-migraine** | 1 (6) |  |

*Notes.* FND = functional neurological disorder; HRT = hormone replacement therapy; NRTI = nucleotide reverse transcriptase inhibitors; CBD = cannabidiol; NSAID=non-steroidal anti-inflammatory drug; PPI = proton pump inhibitor.

| **Variable** | **FND**  **(*n* = 16)** | **Control**  **(*n* = 14)** |  |  |  |
| --- | --- | --- | --- | --- | --- |
|  | **M**  **(*SD*)** | **M**  **(*SD*)** | ***t***  ***(df)*** | ***p*** | ***g*** |
| **HTT Accuracy** | .536  (.239) | .553  (.257) | .19  (26.77) | .85 | .07 |
| **HTT Confidence** | 4.92  (2.46) | 4.95  (2.84) | .04  (25.97) | .97 | .01 |
| **TET Accuracy** | .770  (.136) | .759  (.136) | -.22  (27.47) | .83 | .08 |
| **TET Confidence** | 6.83  (2.04) | 6.71  (1.56) | -.18  (27.54) | .86 | .06 |

**Supplementary Table 2.** Complete case analysis of HTT and TET data.

*Notes.* HTT = Heartbeat Tracking Task; TET = Time Estimation Task; *p<.05, **p<.01, ***p<.001.

**Supplementary Table 3.** Correlations between HTT and TET accuracy for each interval.

| **Interval** | **Group** | **Correlation** |
| --- | --- | --- |
| **23/25s** | **FND** | *r*(14) = .04, *p* = .88 [95% CI = -.46, .53] |
|  | **HC** | *r*(12) = .05, *p* = .87 [95% CI = -.50, .56] |
| **37/35s** | **FND** | *r*(13) = .03, *p* = .93 [95% CI = -.49, .53] |
|  | **HC** | *r*(12) = .53, *p* = .05 [95% CI = .004, .83] |
| **42/45s** | **FND** | *r*(14) = -.05, *p* = .85 [95% CI = -.53, .46] |
|  | **HC** | *r*(12) = .33, *p* = .25 [95% CI = -.25, .73] |

Correlation between raw heartbeats **reported** in HTT and the **number of seconds estimated** in the TET, for each time interval (Desmedt et al., 2020).

**Supplementary Table 4.** Correlations between core outcomes and potentially confounding variables.

| **Variable** | **FND (n=17)** | | **Control (n=17)** | |
| --- | --- | --- | --- | --- |
|  | **r / rho** | **p value** | **r /rho** | **p value** |
| **HTT accuracy** |  |  |  |  |
| TET accuracy | -.27 | .32 | -.07 | .80 |
| Body mass index | .17 | .54 | .11 | .69 |
| Age | .08 | .78 | .09 | .75 |
| Heart rate knowledge | .09 | .74 | .33 | .24 |
| Medication | -.39 | .13 | -.06 | .82 |
| Physical health | -.33 | .21 | .09 | .74 |
| Mental health | .01 | .98 | .29 | .30 |
| Resting heart rate | .16 | .56 | -.19 | .50 |
| PHQ-9 | .20 | .84 | -.22_s_ | .43 |
| PHQ-15 | -.10 | .70 | .05 | .85 |
| GAD-7 | -.42 | .68 | -.02_s_ | .95 |
| FSIQ | .05 | .86 | .26 | .36 |
| MAIA Noticing | -.46 | .09 | -.58 | .046 |
| MAIA Not-Distracting | .29 | .30 | .25 | .44 |
| MAIA Not-Worrying | -.12 | .91 | .30 | .32 |
| MAIA Attention Regulation | -.24 | .40 | -.18 | .56 |
| MAIA Emotional Awareness | -.48 | .08 | -.06 | .86 |
| MAIA Self-Regulation | .23 | .45 | .04 | .89 |
| MAIA Body Listening | -.31 | .33 | .06 | .84 |
| MAIA Trusting | -.09 | .77 | .25 | .40 |
|  |  |  |  |  |
| **HTT confidence** |  |  |  |  |
| TET confidence | .46 | .075 | .31 | .28 |
| Body mass index | -.06 | .83 | .22 | .42 |
| Age | .05 | .84 | -.26 | .35 |
| Heart rate knowledge | .39 | .13 | .26 | .34 |
| Medication | -.12 | .66 | .27 | .33 |
| Physical health | .39 | .13 | .07 | .80 |
| Mental health | -.26 | .33 | .21 | .45 |
| Gender | -.36 | .17 | -.43 | .11 |
| Resting heart rate | .15 | .58 | .21 | .46 |
| PHQ-9 | -.19 | .47 | -.07_s_ | .81 |
| PHQ-15 | .33 | .21 | .17 | .54 |
| GAD-7 | -.09 | .73 | .25_s_ | .37 |
| FSIQ | -.30 | .27 | -.37 | .18 |
| MAIA Noticing | .14 | .63 | -.09 | .77 |
| MAIA Not-Distracting | -.21 | .46 | .13 | .68 |
| MAIA Not-Worrying | -.31 | .27 | .04 | .91 |
| MAIA Attention Regulation | .48 | .08 | .15 | .62 |
| MAIA Emotional Awareness | .29 | .31 | .10 | .74 |
| MAIA Body Listening | .36 | .25 | .18 | .56 |
| MAIA Trusting | .14 | .65 | .29 | .33 |
|  |  |  |  |  |
| **MAIA Not-Distracting** |  |  |  |  |
| Body mass index | .20 | .46 | .22 | .46 |
| Age | -.01 | .98 | -.09 | .75 |
| Heart rate knowledge | .39 | .14 | -.18 | .54 |
| Medication | -.27 | .31 | -.07 | .81 |
| Physical health | -.04 | .90 | -.47 | .09 |
| Mental health | -.33 | .22 | .01 | .96 |
| Gender | -.06 | .81 | -.12 | .68 |
| Resting heart rate | .27 | .31 | .48 | .09 |
| PHQ-9 | -.03 | .91 | .16_s_ | .57 |
| PHQ-15 | -.13 | .63 | .07 | .80 |
| GAD-7 | -.08 | .75 | .29_s_ | .31 |
| FSIQ | -.003 | .99 | .18 | .54 |
|  |  |  |  |  |
| **MAIA Trusting** |  |  |  |  |
| Body mass index | -.25 | .39 | -.33 | .23 |
| Age | .23 | .43 | -.20 | .48 |
| Heart rate knowledge | -.28 | .34 | -.06 | .83 |
| Medication | .25 | .39 | -.10 | .71 |
| Physical health | -.24 | .42 | -.12 | .68 |
| Mental health | -.12 | .68 | .13 | .65 |
| Gender | .28 | .34 | -.25 | .37 |
| Resting heart rate | .04 | .88 | .40 | .17 |
| PHQ-9 | -.20 | .50 | -.52_s_ | .06 |
| PHQ-15 | -.34 | .23 | -.16 | .58 |
| GAD-7 | -.30 | .29 | -.22_s_ | .45 |
| FSIQ | -.37 | .19 | -.32 | .26 |
|  |  |  |  |  |
| **TET accuracy** |  |  |  |  |
| TET confidence | .05 | .85 | -.16 | .55 |

*Notes.* *did not withstand Bonferroni correction; _s_ indicates Spearman’s correlation; HTT = Heartbeat Tracking Task; GAD-7 = Generalized Anxiety Disorder – 7; FSIQ = Full Scale Intelligence Quotient; PHQ-9 = Patient Health Questionnaire – 9; PHQ-15 = Patient Health Questionnaire – 15; TET = Time Estimation Task.

**Supplementary Table 5.** Clinical characteristics by group.

| **Variable** | **FND**  **(n = 17)**  ***M* (*SD*)** | **Control**  **(n = 17)**  ***M* (*SD*)** | ***t***  ***(df)*** | ***p*** | ***g*** |
| --- | --- | --- | --- | --- | --- |
| **AQ** | 20.4 (7.19) | 17.3 (6.91) | -1.26  (31.95) | .11 | .44 |
| **TAS-20** | 53.47 (10.2) | 42.00 (9.97) | -3.32  (31.99) | .001 | 1.11 |
| **MDI-D** | 12.8 (6.68) | 7.47 (2.21) | -3.20  (19.06) | .003 | 1.04 |
| **PHQ-15** | 13.5 (4.02) | 3.24 (2.44) | -8.98  (26.37) | <.001 | 3.01 |
|  | **Median (IQR)** | **Median (IQR)** | ***W*** | ***p*** | ***r*** |
| **GAD-7** | 8 (8) | 2 (4) | 46 | <.001 | .58 |
| **PHQ-9** | 12 (8) | 1 (3) | 16.5 | <.001 | .76 |
| **SDQ-20** | 29 (9) | 20 (0) | 14.5 | <.001 | .79 |
| **MDI-DP** | 6 (9) | 5 (0) | 68 | <.001 | .58 |
| **MDI-DR** | 7 (8) | 5 (0) | 76 | .005 | .49 |
| **MDI-MD** | 7 (5) | 5 (1) | 80 | .023 | .39 |
| **MDI-EC** | 5 (1) | 5 (1) | 134.5 | .70 | .07 |
| **MDI-ID** | 5 (0) | 5 (0) | 119 | .08 | .31 |

*Notes.* AQ = Autism Quotient; GAD-7 = Generalized Anxiety Disorder – 7; MDI-D = Multiscale Dissociation Inventory – Disengagement; MDI-DP = Multiscale Dissociation Inventory – Depersonalisation; MDI-DR = Multiscale Dissociation Inventory – Derealisation; MDI-MD = Multiscale Dissociation Inventory – Memory Disturbance; MDI-EC = Multiscale Dissociation Inventory – Emotional Constriction; MDI-ID = Multiscale Dissociation Inventory – Identity Dissociation; PHQ-9 = Patient Health Questionnaire – 9; PHQ-15 = Patient Health Questionnaire – 15; SDQ-20 = Somatoform Dissociation Questionnaire – 20; TAS-20 = Toronto Alexithymia Scale – 20.

**Supplementary Table 6.** Correlations between interoception and relevant clinical variables in FND (n=17).

| **Variable** | **r / rho** | **p value** |
| --- | --- | --- |
| **HTT accuracy** |  |  |
| SDQ-20 | -.22 | .41 |
| TAS-20 | .21 | .44 |
| AQ | .12 | .66 |
| MDI-D | .05 | .85 |
| MDI-DP | .18_s_ | .51 |
| MDI-DR | .14_s_ | .59 |
| MDI-EC | .43_s_ | .10 |
| MDI-MD | -.02_s_ | .93 |
| MDI-ID | .28_s_ | .30 |
|  |  |  |
| **HTT confidence** |  |  |
| SDQ-20 | -.24 | .37 |
| TAS-20 | -.38 | .14 |
| AQ | -.13 | .63 |
| MDI-D | -.30 | .25 |
| MDI-DP | -.24_s_ | .37 |
| MDI-DR | -.07_s_ | .79 |
| MDI-EC | -.12_s_ | .67 |
| MDI-MD | -.43_s_ | .10 |
| MDI-ID | .01_s_ | .97 |
|  |  |  |
| **MAIA-Not Distracting** |  |  |
| SDQ-20 | -.20 | .47 |
| TAS-20 | .06 | .83 |
| AQ | -.01 | .97 |
| MDI-D | -.09 | .75 |
| MDI-DP | -.25_s_ | .34 |
| MDI-DR | -.33_s_ | .21 |
| MDI-EC | .09_s_ | .74 |
| MDI-MD | -.04_s_ | .87 |
| MDI-ID | -.10_s_ | .71 |
|  |  |  |
| **MAIA-Trusting** |  |  |
| SDQ-20 | -.33 | .25 |
| TAS-20 | .10 | .72 |
| AQ | -.17 | .57 |
| MDI-D | -.54 | .046* |
| MDI-DP | -.30_s_ | .29 |
| MDI-DR | -.42_s_ | .13 |
| MDI-EC | .09_s_ | .76 |
| MDI-MD | -.48_s_ | .08 |
| MDI-ID | -.17_s_ | .57 |

*Notes.* *did not withstand Bonferroni correction; _s_ indicates Spearman’s correlation; AQ = Autism Spectrum Quotient; MDI-D = Multiscale Dissociation Inventory – Disengagement; MDI-DP = Multiscale Dissociation Inventory – Depersonalisation; MDI-DR = Multiscale Dissociation Inventory – Derealisation; MDI-EC = Multiscale Dissociation Inventory – Emotional Constriction; MDI-ID = Multiscale Dissociation Inventory – Identity Dissociation; SDQ-20 = Somatoform Dissociation Questionnaire – 20; TAS-20 = Toronto Alexithymia Scale-20.
